# A STAT3-mediated metabolic switch is involved in tumour transformation and STAT3 addiction

**DOI:** 10.18632/aging.100232

**Published:** 2010-11-15

**Authors:** Marco Demaria, Carlotta Giorgi, Magdalena Lebiedzinska, Giovanna Esposito, Luca D'Angeli, Antonietta Bartoli, Daniel J. Gough, James Turkson, David E. Levy, Christine J. Watson, Mariusz R. Wieckowski, Paolo Provero, Paolo Pinton, Valeria Poli

**Affiliations:** ^1^ Molecular Biotechnology Center and Department of Genetics, Biology and Biochemistry, University of Turin, Via Nizza 52, 10126 Turin, Italy; ^2^ Department of Experimental and Diagnostic Medicine, University of Ferrara, Ferrara, Italy; ^3^ Nencki Institute of Experimental Biology, Department of Biochemistry, Warsaw, Poland; ^4^ Preclinical Imaging Center c/o Center of Molecular Biotechnologies, Bioindustry Park, Colleretto Giacosa, Turin, Italy; ^5^ Department of Pathology and New York University Cancer Institute, New York, NY 10016, USA; ^6^ Department of Molecular Biology and Microbiology, University of Central Florida, Orlando, FL 32826, USA; ^7^ Department of Pathology, University of Cambridge, Cambridge, UK

**Keywords:** cell metabolism, mitochondria, STAT3, tumours, Warburg effect, HIF-1α

## Abstract

The pro-oncogenic transcription factor STAT3 is constitutively activated in a wide variety of tumours that often become addicted to its activity, but no unifying view of a core function determining this widespread STAT3-dependence has yet emerged. We show here that constitutively active STAT3 acts as a master regulator of cell metabolism, inducing aerobic glycolysis and down-regulating mitochondrial activity both in primary fibroblasts and in STAT3-dependent tumour cell lines. As a result, cells are protected from apoptosis and senescence while becoming highly sensitive to glucose deprivation. We show that enhanced glycolysis is dependent on HIF-1α up-regulation, while reduced mitochondrial activity is HIF-1α-independent and likely caused by STAT3-mediated down-regulation of mitochondrial proteins. The induction of aerobic glycolysis is an important component of STAT3 pro-oncogenic activities, since inhibition of STAT3 tyrosine phosphorylation in the tumour cell lines down-regulates glycolysis prior to leading to growth arrest and cell death, both *in vitro* and *in vivo*. We propose that this novel, central metabolic role is at the core of the addiction for STAT3 shown by so many biologically different tumours.

## INTRODUCTION

Signal Transducers and Activators of Transcription (STAT) mediate the signaling downstream of cytokine and growth factor receptors, and several of them play a role in cancer [1,2]. Once activated by tyrosine-phosphorylation, STATs form anti-parallel dimers that concentrate into the nucleus regulating the expression of target genes [3]. STAT3 is activated by cytokines, growth factors and oncogenes [4], and is constitutively tyrosine-phosphorylated in a high percentage of tumours and tumour-derived cell lines of both liquid and solid origin, where its inhibition often triggers growth arrest and/or cell death [1,2,5,6]. Indeed, STAT3 tyrosine phosphorylation and consequent transcriptional activation was shown to be required for cell transformation downstream of several oncogenes, the prototype being v-Src [6-8]. Although STAT3-mediated gene expression signature is mostly consistent with tumour cell survival and proliferation [9,10], it varies in different tumour types, and a core activity determining addiction to STAT3 by a wide spectrum of biologically distinct tumors has not yet been identified [9]. In addition to its canonical nuclear functions, which require tyrosine phosphorylation, DNA binding and transcriptional activity, STAT3 was also reported to exert non-nuclear functions. In particular, it was shown to localize to mitochondria, where it regulates cellular respiration via a yet uncharacterized mechanism [11]. Moreover, we have recently shown that mitochondrial localization requires Serine 727 but not nuclear translocation, DNA binding or tyrosine phosphorylation [12]. This activity, rather than canonical activation, is required for RAS-dependent oncogenic transformation. Thus, STAT3 exerts a central role in mediating tumoural transformation downstream of many different oncogenes and growth factors, via both its canonical transcriptional functions and its non-canonical, non-nuclear activities.

Most tumour cells share the peculiar feature of switching their metabolism towards aerobic glycolysis, i.e. they increase glycolysis and decrease oxidative phosphorylation even in conditions of high oxygen tension [13-15]. This phenomenon, known as the Warburg effect, is thought to lend a metabolic advantage to highly proliferating cells when nutrient supply is not limiting, as it favours the synthesis of essential cellular components required for fast cell duplication. Moreover, pre-adaptation to a glycolytic metabolism is thought to represent an advantage for solid tumours [16], which are often exposed to fluctuating oxygen tension, and reduced cellular respiration may result in lower production of ROS and protection from apoptosis [16-18]. Accordingly, strongly glycolytic tumour cells are critically dependent on glucose, and glycolysis inhibitors have been explored for therapy [19]. The oxygen sensor HIF-1α is a highly unstable protein that becomes stabilized under hypoxia, leading to the activation of glycolysis and the down-regulation of mitochondrial respiration [20,21]. HIF-1α protein level is also increased under normoxia downstream of oncogenes and growth factor receptors via mTor-mediated induction of protein translation, which is known to occur downstream of PI3K activation [22,23], and indeed increased HIF-1α activity is recognized as a major factor contributing to the Warburg effect [21,24-26]. Interestingly, several reports have linked HIF-1α induction with STAT3 activation, proposing either a post-translational or a transcriptional mechanism [27-29]. In particular, H. Yu and co-authors have recently shown that STAT3 activity is required for the hypoxia-induced increase of HIF-1α protein levels downstream of an activated Src oncogene, acting at the level of promoter transcription [28].

We have recently generated knock-in mice expressing physiological levels of the constitutively active STAT3C mutant form [30], and shown its *in vivo* oncogenic potential [31]. In this work we report the analysis of primary mouse embryonic fibroblasts (MEF) derived from *Stat3^C/C^* or *^WT/WT^* embryos. *Stat3^C/C^* cells show an HIF-1α-dependent increased glycolysis and an HIF-1α-independent reduction in mitochondrial respiration. This metabolic switch allows cells to proliferate faster and to be protected from apoptotic and senescence stimuli while becoming highly sensitive to glucose deprivation. Importantly, we can show that STAT3 plays an important role as a master metabolic regulator also in STAT3-dependent human cancer cell lines, offering new insights into its core role as a transcription factor in human cancer.

## RESULTS

### STAT3 constitutive activation elicits pre-oncogenic features in *Stat3*^C/C^ MEFs

**Figure 1.**
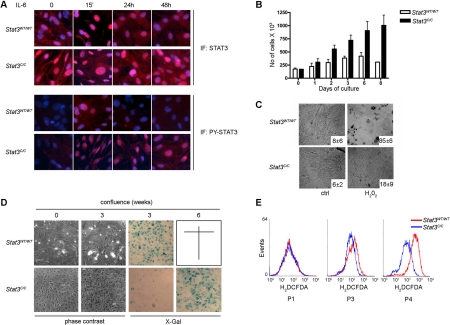
Phenotype of the *Stat3^C/C^* MEFs. Primary MEFs were derived from *Stat3^C/C^* or *Stat3^WT/WT^* embryos and experiments performed on at least three independent samples per genotype. (**A**) Constitutive nuclear localization of STAT3C. Cells of the indicated genotypes were treated or not with IL-6 and stained for total or tyrosine-phosphorylated STAT3. Nuclei are stained in blue with Hoechst. (**B**) Increased growth rates. 1.5*10^5^ cells were plated and counted at the indicated times. Data are mean cell numbers ± s.e.m.. **(C)** Apoptosis protection. Cells were treated with H_2_O_2_ for 16 hours, photographed in phase contrast and stained with Annexin V. Numbers represent the percentage ± s.e.m. of Annexin V positive cells. (**D**) Delayed senescence. Phase contrast: note different viability at 0 and 3 weeks post-confluence. X-Gal: β-galactosidase activity assessed at 3 and 6 weeks post-confluence. *Stat3^WT/WT^* cells were all dead at 6 weeks. (**E)** Decreased ROS production. Cells were regularly passaged and intra-cellular ROS production measured at passage (P) 1, 3 or 4.

We have previously shown that STAT3C displays increased nuclear localization, prolonged activation and enhanced transcriptional activity as compared to the wild-type molecule in MEFs, liver and mammary tumour-derived cells [31]. We confirmed increased localization to the nucleus by immunofluorescence (Figure 1A). Compared to the wild type protein, STAT3C also displays prolonged tyrosine-phosphorylation upon IL-6 treatment, as shown by the enhanced nuclear signal of the phosphorylated form detected 24 and 48 hours after stimulation. *Stat3^C/C^* cells grow faster than their wild type controls (Figure 1B) and display an accelerated cell cycle, observed as a more rapid transit through S-phase (Supplementary Figure S1A). Even though growing as a monolayer, they reach higher cell density at confluence (Figure 1B and 1D, phase contrast) and they are highly resistant to apoptosis induced by treatment with H_2_O_2_ (Figure 1C), starvation, menadione or UV irradiation (Supplementary Figure S1B-E). Moreover, spontaneous senescence is strongly delayed, as shown by beta-galactosidase staining three and six weeks post-confluence (Figure 1D). While by six weeks all *Stat3^WT/WT^* cells were dead, *Stat3^C/C^* cells started to show beta-gal positivity but were able to survive and resume proliferation if passaged (MD, unpublished observation). We then assessed the production of Reactive Oxygen Species (ROS). While ROS accumulation progressively increased with passages in the *Stat3^WT/WT^* cells, it remained constant in the *Stat3^C/C^* cells (Figure 1E). The consequently reduced oxidative stress may account at least partly for the observed resistance to senescence and apoptosis.

### Differential gene expression in the *Stat3^C/C^* and *Stat3^WT/WT^* MEFs

**Figure 2.**
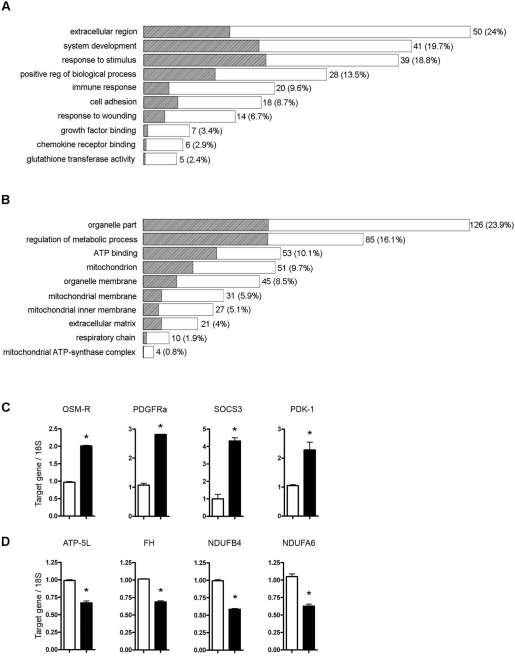
Gene Ontology (GO) analysis on genes differentially expressed in *Stat^WT/WT^* and *Stat3^C/C^* cells. Selected over-represented Gene Ontology functional categories in the lists of genes up- (**A**) and down-regulated (**B**) in the *Stat3^C/C^* versus the *Stat^WT/WT^* cells are shown. The statistical significance of the over-representation was evaluated with a one-sided exact Fisher test. The length of each bar is proportional to the number of differentially expressed genes in the functional category, as indicated by the numbers on the right side, and the shaded portion represents the same number as expected by chance. Numbers in brackets represent the percentage of up- or down-regulated genes that are annotated to the functional category. (**C**) and (**D**) Validation of some microarray data by Taqman RT-PCR quantification of the indicated RNAs. Data are shown as mean values ± s.e.m. of the indicated genes in cells derived from at least three independent embryos per genotype. (**C**) OSM-R, oncostatin M receptor; PDGF-Ra, platelet-derived growth factor receptor; SOCS3, Suppressor of Cytokine Signaling-3; PDK-1, pyruvate dehydrogenase kinase-1. (**D**) ATP-5L, ATP synthase, H+ transporting, mitochondrial F0 complex, subunit G; FH, fumarate hydratase; NDUFB4, NADH dehydrogenase (ubiquinone) 1 beta subcomplex subunit 4; NDUFA6, NADH dehydrogenase (ubiquinone) 1 alpha subcomplex subunit 6. *, p ≤ 0,01. Empty bars or filled bars, *Stat3^WT/WT^* or *Stat3^C/C^* MEFs.

Gene expression profiling revealed about 1000 differentially expressed genes that were organized according to Gene Ontology (GO) annotations (Figure 2A, B). *Stat3^C/C^* MEFs showed significant up-regulation of genes included in GO categories related to known STAT3 functions such as immune regulation, cell adhesion, response to wounding and growth factor binding (Figure 2A, C). Interestingly, down-regulated genes were more represented, and many of them belonged to GO categories related to mitochondrial function (Figure 2B, D and Supplementary Figure S2). Conversely, several genes involved in glycolysis were highly expressed in the *Stat3^C/C^* cells including pyruvate dehydrogenase kinase-1 (Pdk-1). PDK-1 is a key glycolysis regulator that acts by inactivating the mitochondrial pyruvate dehydrogenase (PDH) complex [24], thus limiting the amount of pyruvate entering the citric acid cycle (Figure 2C).

**Figure 3.**
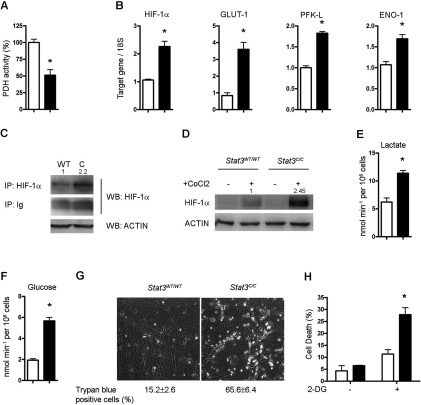
Glycolytic metabolism of *Stat3^C/C^* MEFs. The histograms represent mean values ± s.e.m. of three independent experiments. Empty bars or filled bars, *Stat3^WT/WT^* or *Stat3^C/C^* MEFs respectively. *, p < 0,01. (**A**) Histograms show the pyruvate dehydrogenase (PDH) activity expressed as percentage of that detected in the *Stat3^WT/WT^* MEFs. (**B**) Taqman RT-PCR quantification of HIF-1α, hypoxia-inducible factor-1α; GLUT-1, glucose transporter-1; PFK-L, phospho-fruktokinase-liver type; ENO-1, enolase-1. (**C,D**) HIF-1α protein quantification. (**C)** Immunoprecipitation followed by Western blot of total protein extracts with anti-HIF-1α antibodies. ACTIN was quantified in the total extracts as a loading control. The numbers at the top of the lanes represent the quantification of the HIF-1α-specific signals upon normalization to IgGs. (**D**) Western blot. Cells were treated or not with Cobalt Chloride (CoCl_2_) for 4 hours and nuclear protein extracts were stained for HIF-1α and ACTIN as an internal control. The numbers at the top of the lanes represent the quantification of the HIF-1α-specific signals upon normalization to ACTIN. (**E**) Lactate production was measured in the culture medium as a function of concentration, time and cell number. (**F**) Glucose intake was calculated as the difference in glucose concentration in the medium before and after cell culturing. (**G,H**) Increased sensitivity of *Stat3^C/C^* MEFs to glucose deprivation. (**G**) Cells were grown for 48 hours in medium with no glucose and cell viability evaluated by trypan blue staining. Numbers show the percentage ± s.e.m. of trypan blue positive cells. (**H**) Cells were treated for 48 hours with the glucose analogue 2-DG. Cell death was measured by flow cytometry and represented as the portion of cells in the sub G1/G0 region upon propidium iodide staining.

### *Stat3^C/C^* MEFs display features of aerobic glycolysis

In agreement with the observed Pdk-1 up-regulation, PDH activity in *Stat3^C/C^* cells was reduced by about 50% (Figure 3A). *Pdk-1* is a known target of the hypoxia inducible factor (HIF)-1α[32], which in turn can be transcriptionally induced by STAT3 [27]. Indeed, *Stat3^C/C^* MEFs show significantly increased Hif-1α mRNA expression (Figure 3B). HIF-1α protein levels were also elevated, as shown by immunoprecipitation of whole cell extracts with anti-HIF-1α antibodies followed by Western blot (Figure 3C). Accordingly, we also detected up-regulation of several known HIF-1α target genes [21] such as the glucose transporter Glut-1 and two key glycolytic enzymes, phospho-fruktokinase L-type (Pfk-L) and enolase-1 (Eno-1) (Figure 3B). In agreement with the observed increased expression of HIF1-α and of several of its targets encoding proteins involved in glycolysis, *Stat3^C/C^* cells exhibit a glycolytic phenotype, producing higher amounts of lactate (Figure 3E) and enhancing their glucose intake (Figure 3F). Accordingly, they are highly sensitive to glucose deprivation as compared to their wild type controls, as shown both by cultivating cells in glucose-free medium (Figure 3G) and by treating them with *2-Deoxy-D-glucose* (2-DG), a glucose analogue that inhibits glycolysis [19] (Figure 3H). These features are highly reminiscent of the well-known Warburg effect, or aerobic glycolysis, shared by most cancer cells [13,15].

**Figure 4.**
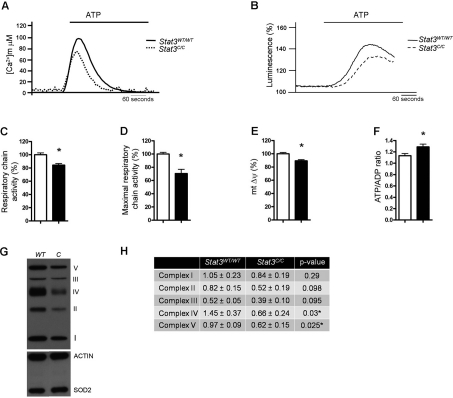
Decreased mitochondrial activity and enhanced ATP/ADP ratio of *Stat3^C/C^* MEFs. (**A**) Mitochondrial Ca^2+^ homeostasis. MEFs of the indicated genotypes were transduced with a mitochondria-targeted aequorin (AEQ), which was measured then upon challenging with 100 μM ATP as indicated. (**B**) ATP-induced changes in ATP concentration in mitochondria. MEFs were transiently transfected with a mitochondria-targeted luciferase 36 hours prior to ATP measurement, and data expressed as a percentage of the initial value. (**A,B**) Data are representative of at least 10 traces, each from 3 independent experiments. (**C**) Respiratory chain activity measured with resazurine. *, p < 0.01 (n=6). (**D**) Maximal respiratory chain activity, measured with the use of resazurine in the presence of 300nM FCCP. *, p < 0.01 (n=6). (**E**) Mitochondrial membrane potential. *, p < 0.05 (n=9). (**C-E**) Data are mean ± s.e.m., expressed as percentage of the value detected in the *Stat3^WT/WT^* MEFs. (**F**) ATP/ADP ratio was expressed as mean ± s.e.m.of four independent samples per genotype. *, p < 0.05. (**C-F**) Empty bars or filled bars, *Stat3^WT/WT^* or *Stat3^C/C^* MEFs respectively. (**G**) Western blot with antibodies against specific ETC components. CI subunit NDUFB8, complex I; CII-30kD, complex II; CIII-Core protein, complex III; CIV subunit, complex IV; CV alpha subunit, complex V. Actin and SOD2 were used as internal control for total and mitochondrial content. (**H**) Quantification of the different complexes, shown as mean ± s.e.m. of three independent samples per genotype. P values are shown.

### Reduced mitochondrial activity in *Stat3^C/C^* MEFs

**Figure 5.**
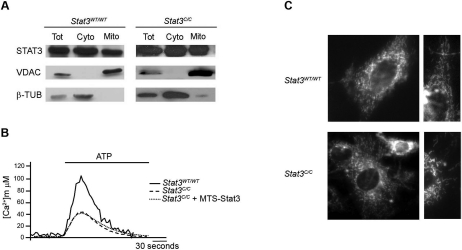
STAT3-mitochondrial localization and mitochondria morphology. (**A**) Intracellular localization of Stat3. Sub-cellular fractions were isolated and protein extracts were prepared as described in M&M. Western blot analysis was performed using antibodies against STAT3, and VDAC-1 or β-TUBULIN were used as mitochondrial and cytoplasmic markers, respectively. Tot, whole unfractionated extract; Cyto, cytoplasmic fraction; Mito, mitochondrial fraction. (**B**) Effects of expressing a mitochondria targeted STAT3 form (MTS-Stat3) on mitochondrial Ca^2+^ homeostasis. MEFs were co-transfected with mitochondria-targeted aequorin (AEQ) and MTS-Stat3, and AEQ measured upon challenging with 100 μM ATP. Traces are representative of at least 10 from 3 independent experiments yielding similar results. (**C**) Mitochondrial morphology was visualized by loading MEFs with 10 nM TMRM. The field of cells is representative of > 50 observations from 2 independent experiments.

Cancer cells displaying aerobic glycolysis also co-ordinately down-regulate cellular respiration, although the mechanism is not entirely clear [33]. The significant down-regulation of nuclear-encoded genes involved in mitochondrial function observed in the *Stat3^C/C^* MEFs (Supplementary Figure S2), together with their decreased PDH activity (Figure 3A), may lead to the reduction of mitochondrial respiration. In order to assess this, we decided to compare mitochondrial-specific Ca^2+^ uptake and mitochondrial ATP production in the *Stat3^C/C^* and *Stat3^WT/WT^* cells. It is indeed well known that the mitochondrial membrane potential acts as the driving force for the transporter responsible for mitochondrial Ca^2+^ uptake [34]. Inhibition of the respiratory chain, which in turn reduces the mitochondrial membrane potential, abolishes the ability of mitochondria to accumulate Ca^2+^, making Ca^2+^ influx a well accepted measure of mitochondrial activity [35-37]. *Stat3^C/C^* MEFs showed reduced Ca^2+^ uptake upon ATP stimulation (Figure 4A). Accordingly, both mitochondrial ATP production and basal respiratory chain activity were reduced in the *Stat3^C/C^* MEFs (Figure 4B, C). This correlated with lowered maximal respiratory chain activity (measured in the uncoupled state) and slightly reduced mitochondrial membrane potential (Figure 4D, E), which in turn may explain the diminished ROS production observed in the *Stat3^C/C^* MEFs (see Figure 1E). Moreover, in agreement with the microarray data, the protein levels of representative components of the Electron Transport Chain (ETC), particularly those belonging to complexes IV and V, were reduced in the *Stat3^C/C^* cells (Figure 4G). Taken together, these data demonstrate that *Stat3^C/C^* MEFs feature a reduction of their mitochondrial metabolism, caused at least in part by the lower expression of ETC components. Despite their lower ATP production, *Stat3^C/C^* cells show an increased ATP:ADP ratio (Figure 4F), suggesting a favourable energy balance similar to that observed in glycolytic tumour cells and able to support their increased proliferation rates. It could be argued that the STAT3C mutant might display defective mitochondrial functions, which in turn may affect mitochondrial activity in the *Stat3^C/C^* MEFs. Several lines of evidence suggest however that STAT3C mitochondrial functions are unaffected, and thus that the reduced mitochondrial activity of the *Stat3^C/C^* MEFs is likely a direct effect of STAT3C constitutive transcript-tional activity. First, the mitochondrial localization of STAT3C was indistinguishable from that of the wild type protein, as shown by fractionation experiments (Figure 5A). Second, ectopic expression of mitochondria-targeted STAT3 (MTS-STAT3), which normalized the defective respiration of RAS-transformed *Stat3^-/-^* MEFs [12], could not rescue mitochondrial Ca^2+^-uptake in the *Stat3^C/C^* MEFs (Figure 5B). Finally, both mitochondrial morphology and mass were normal in the Stat3^C/C^ MEFs (Figure 5C and not shown), as were the levels of Hif-1α, Pdk-1 and lactate in *Stat3*^-/-^ MEFs (Supplementary Figure S3D, E). Since neither nuclear nor mitochondrial STAT3 are required to maintain basal glucose metabolism and HIF-1a levels, the observed mitochondrial phenotype cannot be caused by a defective mitochondrial or nuclear function of the STAT3C protein.

**Figure 6.**
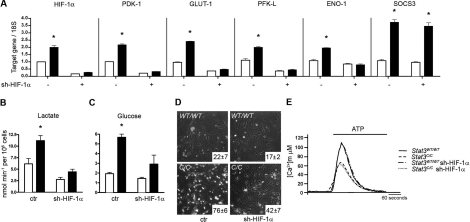
HIF-1α silencing normalizes glycolytic metabolism but not mitochondrial activity of *Stat3^C/C^* MEFs. Empty bars or filled bars, *Stat3^WT/WT^* or *Stat3^C/C^* MEFs respectively, either silenced or not for HIF-1α (sh-HIF-1α), represent mean values ± s.e.m. of three independent experiments. *, p ≤ 0,001. (**A**) Taqman RT-PCR quantification of the indicated mRNAs. (**B-D**) Lactate production, glucose intake and sensitivity to glucose deprivation were measured as described in the legend to Fig. 3. (**E**) Mitochondrial Ca^2+^ homeostasis, measure as described in the legend to Figure 4.

### Hif-1αis responsible for the induction of aerobic glycolysis but not for the reduced mitochondrial activity of *Stat3^C/C^* cells

The up-regulation of HIF-1α observed in the *Stat3^C/C^* cells appears to occur mainly via increased expression rather than protein stabilization, since treatment with the iron chelator CoCl_2_, which blocks HIF-1α degradation, triggered much higher protein accumulation in the *Stat3^C/C^* cells than in the wild type counterparts (Figure 3D). Another well-known mechanism of HIF-1α induction is the mTOR-dependent enhanced translation occurring downstream of PI3K activation [22,23]. PI3K did not however appear to be involved in this context, since its inhibition could not affect either the expression of Hif-1α and Pdk-1, or the production of lactate (Supplementary Figure S3A-C). Therefore, STAT3-mediated induction of Hif-1α mRNA levels seems to fully account for its increased expression.

Interestingly, the silencing of Hif-1α normalized the glycolytic metabolism of *Stat3^C/C^* MEFs, down-regulating Pdk-1, Glut-1, Pfk-L and Eno-1 mRNAs but not the glycolysis-unrelated STAT3 target Socs3 (Figure 6A). Accordingly, lactate production, glucose intake and sensitivity to glucose deprivation were significantly reduced (Figure 6B-D). The expression of STAT3C, which mimics the constitutive STAT3 activation observed in many tumours, is thus sufficient to promote aerobic glycolysis, acting at least in part through transcriptional induction of Hif-1α. Of note, Hif-1α silencing lowered the expression levels of the Hif-1α target genes as well as the production of lactate and of glucose intake also in the *Stat3^WT/WT^* MEFs, suggesting that Hif-1α plays a role in promoting basal levels of glycolysis also in wild type cells.

In contrast to the glycolytic metabolism, which was entirely dependent on Hif-1α, the mitochondrial Ca^2+^-uptake by *Stat3^C/C^* cells was completely unaffected by Hif-1αsilencing and consequent Pdk-1 down-regulation (Figure 6E and data not shown). Additionally, the silencing of Hif-1α could not rescue the expression of nuclear genes encoding for mitochondrial proteins (Supplementary Figure S2B). These data clearly demonstrate that the up-regulation of glycolysis and the down-regulation of mitochondrial function of *Stat3^C/C^* MEFs, both mediated by constitutively transcriptionally active STAT3, occur via independent pathways. The leading cause of reduced mitochondrial activity appears to be the STAT3-mediated down-regulation of nuclear genes encoding for mitochondrial proteins, mirrored by the lowered expression of ETC components.

**Figure 7.**
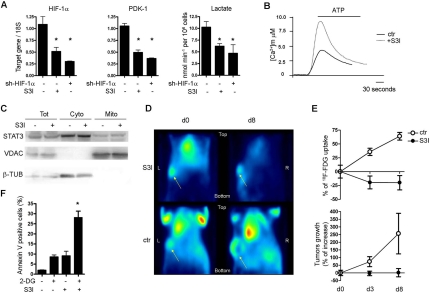
*In vitro* and *in vivo* STAT3-dependent glycolytic metabolism in MDA-MB468 human breast tumour cells. (**A**) Expression of the indicated mRNAs (by Taqman RT-PCR) and lactate production were measured in MDA-MB468 cells, either treated with the S3I STAT3 inhibitor for 12 hours or silenced for HIF-1α, as indicated. Data are shown as mean values ± s.e.m. of three independent experiments. *, p ≤ 0,001. (**B**) The mitochondrial Ca^2+^ response was assessed as described in the legend to Figure 4, in cells either treated or not with S3I for 12 hours. (**C**) STAT3 sub-cellular localization was assessed as described in the legend to Figure 5. (**D**) Tumour ^18^F-FDG uptake. Mice were inoculated with MDA-MB468 cells and tumours let grow up to 60 mm^3^ prior to S3I and ^18^F-FDG treatment. Images were acquired at the indicated times after the first S3I treatment. Shown are coronal section of tumour of one (out of five) S3I-treated (8 days) and one (out of three) control mice. Yellow arrows indicate the tumours. (**E**) The upper graph represents the variation of glucose uptake normalized over tumour size at the indicated times after starting S3I treatment. % of ^18^F-FDG uptake= (Suv_d=n_ - Suv_d=0_)*100/ Suv_d=0_. The lower graph represents the mean tumour volume ± s.e.m. at the same times. Note decreased glucose uptake at day 3 (d3) and 8 (d8) upon S3I treatment, compared to constant tumour volume. (**F**) Co-operativity between glucose deprivation and S3I treatment. Cells were treated for 48 hours with the glucose analogue 2-DG and S3I, either alone or in combination, at sub-optimal concentrations. Data are shown as the percentage ± s.e.m. of Annexin V positive cells. *, p < 0,05 (n=4).

### STAT3-addicted tumour cell lines undergo STAT3-dependent aerobic glycolysis

Our data suggest that constitutively active STAT3 can act as a central mediator of aerobic glycolysis, which would explain the general STAT3 addiction of cancer cells. To test this idea, we assessed the effects of inhibiting STAT3 on the glycolytic metabolism and mitochondrial activity of three STAT3-dependent epithelial tumour cell lines, MDA-MB468, SKBR3 and DU145, all of which display constitutively active STAT3 [38]. Inhibition of STAT3 activity with the S3I compound [2], which interferes with the STAT3 SH2 domain and consequently with STAT3 tyrosine phosphorylation and transcriptional activity, causes massive apoptosis after 24 hours, but 12 hours treatment is sufficient to strongly down-regulate constitutive STAT3 phosphorylation avoiding cell death (Supplementary Figure S4A, B). In all cell lines, 12 hours S3I treatment dramatically lowered Hif-1α and Pdk-1 expression and decreased lactate production (Figure 7A and Supplementary Figure S4B, C), at the same time rescuing mitochondrial-Ca^2+^ uptake (Figure 7B and Supplementary Figure S4B, C). As expected, mitochondrial STAT3 localization, known to be independent of tyrosine phosphorylation, was not modified (Figure 7C). Gene expression, lactate production and mitochondrial-Ca^2+^ uptake were completely unaffected by STAT3 inhibition in T47D cells, which do not display constitutively active STAT3 and are insensitive to STAT3 inhibition (Supplementary Figure S4A, D). Thus, tumour cell lines with constitutive STAT3 phosphorylation and dependent on STAT3 for survival exhibit a strictly STAT3-dependent aerobic glycolytic phenotype, comparable to that observed in the *Stat3^C/C^* MEFs.

Similar to what observed in the *Stat3^C/C^* MEFs, Hif-1α silencing down-regulated Pdk-1 expression and lactate production but not mitochondrial-Ca^2+^ uptake in MDA-MB468 cells (Figure 7A and data not shown), suggesting that also wild type STAT3, when constitutively activated in cancer, can induce aerobic glycolysis via both HIF-1α-dependent and -independent mechanisms.

### STAT3-dependent glycolysis also occurs *in vivo*

To confirm the fundamental role of STAT3 in regulating the glycolytic switch of STAT3-dependent tumour cells *in vivo*, glucose uptake by xenografted MDA-MB468 tumours was measured in the presence or absence of the S3I treatment by means of PET analysis using the radioactive glucose-analogue ^18^F-FDG (PET-FDG, Figure 7D). Treatment was started when the tumours had reached the volume of 80 mm^3^ (day 0). The tumours of control mice displayed higher increase in glucose uptake than in tumour volume, as shown by the sharply enhanced FDG signal even upon normalization to tumour size (Figure 7E, upper panel). In contrast, tumour growth was arrested and glucose uptake reduced upon S3I treatment already at 3 days (Figure 7E), suggesting that inhibition of STAT3 activity has prominent effects on glucose metabolism also *in vivo*. Interestingly, treatment of MDA-MB468 cells with a combination of S3I and 2-DG at sub-optimal doses yielded cooperative effects on cell apoptosis, suggesting the potential therapeutic advantage of combining glucose deprivation and STAT3 inhibition (Figure 7F).

## DISCUSSION

**Figure 8.**
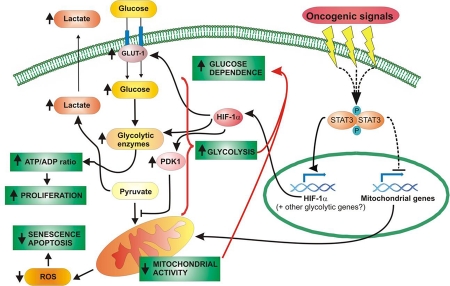
STAT3 acts as a central mediator of cell metabolism through both HIF-1α-dependent and -independent mechanisms. Many oncogenic signals can trigger the constitutive activation of STAT3, either directly or indirectly. Activated STAT3 migrates into the nucleus, where it up-regulates HIF-1α expression and lowers the expression of mitochondrial mRNAs, either via direct or indirect mechanisms. HIF-1α induces the transcription of different genes involved in glycolysis; the glucose channel GLUT-1 enhances glucose intake; the kinase PDK-1 reduces the conversion of pyruvate into Acetyl-CoA, favouring its catabolism into lactate; other enzymes, such as ENO-1 or PFK-L, sustain glycolysis by improving glucose metabolism. Increased glycolysis results in enhanced lactate production, and allows the cell to maintain a high ATP/ADP ratio even in the presence of reduced mitochondrial respiration. All together, this results in enhanced proliferative potential. The decreased mitochondrial activity, insensitive to HIF-1α silencing, is instead predominantly caused by the down-regulation of nuclear-encoded mitochondrial genes and leads to reduced oxidative metabolism, which in turn prevents ROS over-production protecting the cell from senescence and apoptosis. The metabolic switch from oxidative phosphorylation to aerobic glycolysis, typical of most cancer cells, makes cells highly sensitive to glucose deprivation.

Most cancer cells share the feature of metabolizing glucose by aerobic glycolysis -the Warburg effect- and the inducible α subunit of the HIF-1 transcription factor lies at the crossroad of both anaerobic and aerobic glycolysis [26]. Indeed, HIF-1α induces all know glycolysis-related genes while down-regulating mitochondrial activity via PDK-1 [21]. HIF-1α activity is thought to be controlled mainly at the protein level, being highly unstable under normal oxygen tension since it is continuously synthesized and then modified via prolyl hydroxylation followed by von-Hippel-Lindau (VHL)-mediated proteasomal degrada-tion [39]. Hypoxic conditions decrease the activity of prolyl hydroxylases (PHDs), thus inhibiting the interaction with VHL and resulting in protein stabilization [40]. On the other hand, growth factors and oncogenes can also increase HIF-1α activity via enhanced protein translation mediated by PI3K-induced mTOR [22,23]. STAT3 has been proposed to contribute to HIF-1α protein stabilization either via Akt activation [27] or via interaction with VHL and consequent inhibition of VHL-HIF-1α interaction [29]. Recently, however, STAT3 was shown to enhance HIF-1α RNA transcription under hypoxia, since it was required to mediate HIF-1α up-regulation (both protein and RNA) upon hypoxic stimulation of v-Src-transformed cells, and was able to bind to the *Hif-1*α promoter [28]. For the first time we show here that even under normoxia STAT3 constitutive transcriptional activity is sufficient to induce a two-fold increase in Hif-1α mRNA levels, in turn resulting in similarly higher protein levels. The need for constitutive activation is built into the intrinsic instability of the HIF-1α sensor, and is likely to represent an important functional difference between acute and constitutive STAT3 activity. As mentioned in the results section (Figure 3D and Supplementary Figure S3A-C), neither protein stabilization nor PI3K-mediated translation enhancement appear to play a role in the higher HIF-1α levels detected in the Stat3^C/C^ MEFs. This relatively low HIF-1α induction is sufficient (and necessary, as shown by the silencing experiments) to drive a metabolic switch to aerobic glycolysis, i.e. the Warburg effect. Interestingly, while under hypoxic conditions HIF-1α actively down-regulates mitochondrial activity via PDK-1 induction, the increase in PDK-1 detected in the *Stat3^C/C^* MEFs is not apparently involved in their reduced mitochondrial activity, which cannot be rescued by Pdk-1 normalization upon Hif-1α silencing. Thus, as depicted in Figure 8, constitutive STAT3 activity, occurring in a wide variety of tumours downstream of many oncogenic signals, is sufficient to determine the switch to aerobic glycolysis via two distinct nuclear mechanisms: i) the induction of Hif-1α transcription, which in turn up-regulates genes involved in glycolysis. This allows fast proliferation and highly increases glucose consumption, leading to glucose dependence, just like all known glycolytic cancer cells; ii) the down-regulation of mitochondrial activity, which is HIF-1α- and PDK-1-independent and apparently caused by the STAT3-mediated reduced expression of many nuclear genes encoding for mitochondrial proteins, leading to reduced levels of ETC components. At present, we do not know if this is due to a direct effect of STAT3 on their transcription, or, more likely, to the indirect regulation of a common repressor or a targeting microRNA(s). The reduced mitochondrial activity may contribute to the decreased ROS accumulation observed in the *Stat3^C/C^* MEFs, which in turn is likely to trigger the high resistance of these cells to apoptosis and senescence, two hallmarks of cellular transformation.

STAT3 emerges as a central player in determining the switch to aerobic glycolysis, and this in turn can explain why so many biologically distinct tumours are addicted to its activity for continuous survival and growth even though sometimes do not strictly require it for transformation [41]. It is indeed well known that tumour cells displaying the Warburg effect become addicted to high glucose influxes, and that enhancing aerobic glycolysis can favour tumoural transformation. This idea is corroborated by the observation that several tumour cell lines previously shown to be strictly STAT3-dependent present a phenotype super-imposable to that of the *Stat3^C/C^* MEFs, with high glycolysis levels and low mitochondrial respiration, both mediated by STAT3 transcriptional activity. Indeed in these cells, but not in similar cells not displaying constitutive STAT3 activation and accordingly independent of STAT3 for survival, inhibition of STAT3 activity normalizes glycolysis prior to leading to apoptotic cell death, suggesting that STAT3 addiction is at least partly linked to STAT3-induced aerobic glycolysis. Exactly as observed in the *Stat3^C/C^* MEFs, while enhanced glycolysis is dependent on HIF-1α, mitochondrial respiration is unaffected by HIF-1α silencing. Importantly, the observation that treatment with the S3I STAT3 inhibitor lowers glucose uptake by tumours prior to arresting their growth, suggests that a similar mechanism for STAT3 addiction occurs *in vivo* as well. It is puzzling why cancer cells should specifically become dependent on STAT3 for aerobic glycolysis, since most STAT3-activating oncogenic signals can also activate PI3K, a known mediator of this phenomenon. Possibly, STAT3 activity is more specific/less dispensable since it can at the same time regulate glycolysis and mitochondria. Alternatively, even when not the only factor inducing the Warburg effect, its contribution may nevertheless be crucial. Further studies will be required to clarify this issue.

Taken together with the metabolic role of mitochondrial STAT3 recently reported by us and others [11,12], STAT3 emerges as a central regulator of cell metabolism in both transformed and non-transformed cells, acting both in the nucleus and in mitochondria. In the nucleus, as shown here, STAT3 constitutive activation/tyrosine phosphorylation, which is known to occur downstream of many oncogenic pathways, promotes aerobic glycolysis and reduces mitochondrial respiration without affecting mitochondrial mass or morphology. This activity is likely to account for the addiction to STAT3 observed in many tumours, displaying a variety of abnormally activated oncogenic pathways that share the ability to induce STAT3 tyrosine phosphorylation and aerobic glycolysis. In contrast to its nuclear counterpart, mitochondrially localized STAT3 is not phosphorylated on tyrosine 705, the hallmark of transcriptional activation, but on Serine 727, promoting oxidative phosphorylation in both non transformed pro-B cells [11] and Ras-transformed MEF cells [12]. Moreover, it favours aerobic glycolysis downstream of Ras oncogenes, which trigger Serine-STAT3 phosphorylation, and this activity is required for Ras-mediated transformation [12]. Although the roles played by nuclear or mitochondrial STAT3 may seem contradictory, it must be borne in mind that specific phosphorylation on tyrosine or serine occurs upon distinct stimuli and under distinct physiological or pathological conditions, leading to two functionally distinct molecules. Our results suggest indeed that it will be important to distinguish between the nuclear and mitochondrial actions of STAT3 when designing STAT3 inhibitors for therapeutic applications.

We propose that this central metabolic role played at multiple levels may be at the core of the addiction for STAT3 shown by so many biologically different tumours. In addition, it may also contribute to the protective role described for this factor in tissue damage following ischemia-reperfusion or heart infarction [42,43].

Finally, our data suggest that a combination of STAT3 inhibition with glucose deprivation may represent a valuable therapeutic strategy in cancer, providing a mean to hit fundamental metabolic functions of a wide variety of STAT3-dependent, highly glycolytic tumours more effectively than STAT3 inhibition alone.

## METHODS

### Mice, MEFs preparation and culture, cell lines and treatments

*Stat3^C/C^* mice [31] were maintained in the transgenic unit of the Molecular Biotechnology Center (University of Turin). Procedures were conducted in conformity with national and international laws and policies as approved by the Faculty Ethical Committee. Embryos were dissected 13.5 days post coitum for MEF derivation. Primary MEFs, 3T3 MEFs [44], MDA-MB468 and SKBR3 cells (ATCC, Manassas VA, USA) were grown in DMEM with GLUTAMAX (Dulbecco's modified Eagle medium; Gibco-BRL, Carlsbad CA, USA), DU145 (ATCC) were grown in RPMI 1360 (Gibco-BRL). Both media were supplemented with 10% (v/v) heat-inactivated FCS (fetal calf serum; Gibco-BRL), 100 U/ml penicillin, 100 μg/ml streptomycin. Treatments: S3I-201 inhibitor [2]. 100 μM (optimal dose) or 50 μM (sub-optimal dose) in DMSO for 12 and 24 hours; Cobaltous chloride hexahydrate (Sigma Aldrich, St. Louis MO, USA), 500 μM for 4 hours; Ly294002 PI3-K inhibitor, 40 μM for 48 hours.

### Proliferation rate and cell cycle analysis

For proliferation rate, 1.5*10^5^ cells were seeded in 6-well plates and counted at the indicated times using the Countess Automated Cell Culture (Invitrogen, Carlsbad CA, USA).

For cell cycle analysis, sub-confluent cells were starved 24h hours, re-stimulated with 10% FCS, detached and stained with propidium iodide solution (2.5 mg/ml PI (Sigma Aldrich), 0.1 mg/ml RNaseA, 0.05% Triton X-100) at the indicated times, followed by flow cytometry analysis.

### In vitro cell death, senescence and ROS production

Cell death: cells were treated with Menadione (Sigma Aldrich, 7.5 μM for 24 hours), H_2_O_2_ (Sigma Aldrich, 1 mM for 16 hours), irradiated with 10 J/m^2^ UV-C or serum-starved for 72 hours, followed by staining with either with trypan blue, Annexin-V, anti-activated Caspase-3 or by Tunel assay. Senescence: cells were stained at the indicated times after plating using a Senescence Cells Histochemical Staining Kit (Sigma Aldrich), according to manufacturer's protocol. ROS measurement: equal numbers of cells were incubated with 5 μM H_2_DCFDA (Molecular Probes, Invitrogen) for 30 min at room temperature and analyzed by flow cytometry.

### Microarray analysis

Micro array data are accessible from the Gene Expression Omnibus (http://www.ncbi.nih.gov/geo/) under accession GSE21507.

Total RNA was prepared from sub-confluent MEF cells derived from three independent embryos per genotype. Samples were analyzed using the MouseWG-6 v 1.1 Expression BeadChip (Illumina, San Diego CA, USA) as previously described [31]. Briefly, RNA was first reverse transcribed using oligo (dT) primers to synthesise first strand cDNA, followed by second strand synthesis. cDNA was then purified to remove salt, RNA, enzymes, and excess primers. Subsequent *in vitro* transcription synthesised biotin-labelled cRNA, which was further purified and then hybridised to the array chip.

### Real Time-PCR

Total RNA was prepared with the PureLink Micro-to-Midi total RNA Purification System (Invitrogen). qRT-PCR reactions were performed as previously described [44], using the Universal Probe Library system (Roche Italia, Monza, Italy). The 18S rRNA pre-developed TaqMan assay (Applied Biosystems) was used as an internal control. For primers and probes see Supplementary Information.

### Lentiviral infection

pLKO vectors carrying either scrambled or shRNA-HIF-1α sequences (Open Biosystems, Huntsville AL, USA) were packaged by transfecting 293T cells and used to infect cells for 24 hours, followed by puromycin selection for 48 hours.

### FACS Analysis

H_2_DCFDA and Annexin-V emission were detected in the green channel (525 nm) and propidium iodide in the red channel (575 nm) following excitation by a 488 nm laser on a FACS Calibur cytometer (Beckton, Dickinson and Company, Franklin Lakes NJ, USA).

### Western blot

Total, nuclear, mitochondrial and cytosolic protein extracts, obtained as previously described [45] were fractionated on SDS-PAGE and transferred to a polyvinylidene difluoride membrane (Millipore, Billerica MA, USA).

### Glucose and lactate measurements

Glucose or lactate were measured in cell supernatants 3 hours after changing medium using a Glucose Assay Kit (Sigma Aldrich) or a Lactate Colorimetric Assay Kit (Abcam). Data were normalized to final cell counts. Glucose intake was calculated as the difference in glucose concentration between fresh medium and supernatant.

### Glucose dependence

For glucose deprivation, cells were cultivated in DMEM containing 0 g/l of glucose and 3% FBS for 48 hours, then stained with Trypan Blue (Invitrogen). For 2-deoxyglucose (2-DG) treatment, cells were treated with 1.5 mg/ml (MEFs) or 1 mg/ml (MDA-MB468) of 2-DG (Sigma Aldrich) for 48 hours, then stained with propidium iodide and/or AnnexinV followed by flow cytometry analysis.

### Calcium and ATP measurements

Cells were grown on glass coverslips at 50% confluence. For Ca^2+^ measurements, cells were infected with the adenovirus expressing the appropriate aequorin chimera as previously described [46]. Measurements were carried out in KRB (125 mM NaCl, 5 mM KCl. 1 mM MgSO_4_, 1 mM Na_2_HPO_4_. 5.5 mM glucose, 20 mM NaHCO_3_, 2 mM l-glutamine and 20 mM HEPES pH 7.4, supplemented with 1 mM CaCl_2_). Agonists and other drugs were added to the same medium. Cells were lysed with 100 μM digitonin in a hypotonic Ca^2+^-rich solution (10 mM CaCl_2_ in H_2_O), thus discharging the remaining aequorin pool. The light signal was collected and calibrated into [Ca^2+^] values, as previously described [46].

For measuring mitochondrial ATP, MEFs were transfected withmitochondrial luciferase (mtLuc), and luminescence measured after 36 hours as previously described [47]. Cells were constantly perfused with a modified KRB containing 20 μM luciferin (Sigma Aldrich).

### Immunofluorescence

Cells plated on glass coverslips were washed in PBS, fixed in 4% paraformaldehyde, quenched with 50 mmol/L ammonium chloride, permeabilized with 0.3% Triton X-100 in PBS, saturated with 3% bovine serum albumin, and incubated with primary antibodies at room temperature for 1 h, followed by fluorescein-labeled secondary antibodies (Sigma Aldrich) and then by Hoechst-dye. Tunel assay (Roche Diagnostic Corp., Indianapolis IN, USA) was performed according to manufacturer's protocol. An Axiovert 200M Zeiss microscope or the Axio-Observer-Z1 Zeiss microscope with the ApoTome system for optical sectioning were used. Images were acquired with MetaMorph software (Molecular Devices, Toronto, Canada) or the AxioVision release 4.6.3 software (Carl Zeiss, Inc., Oberkochen, Germany), respectively.

### PDH activity

10^6^ cells were plated on a 100*15 mm dish and detached after 24 hours. PDH activity was measured using the PDH mitoprofile kit (Invitrogen) according to manufacturer's protocol.

### Immunoprecipitation

Freshly prepared pre-cleared lysates were incubated O/N at 4°C with anti-HIF-1α antibody and 20 μl of proteinG-Sepharose beads (Ge Healthcare Bio-Science, Uppsala, Sweden). Immunoprecipitated proteins were boiled in 1x Laemmli buffer for 5 min.

### Mitochondrial membrane potential (mtΔΨ)

Cells grown in 24-well plates were incubated with 10 μM JC1 (5,5',6,6'-tetrachloro-1,1',3,3'-tetraethyl-benzimidazolylcarbocyanine iodide) in PBS containing 5 mM glucose for 10 min at 37°C followed by fluorescence recording in a microplate reader (Infinite M200, Tecan, Austria) at 485 nm excitation/520 nm emission and 535 nm excitation/635 nm emission wavelengths.

### Respiratory chain activity

MEFs grown in 24-well plates were washed with PBS, PBS containing 5 mM glucose and 6 μM resazurine was added and fluorescence was recorded immediately in a microplate reader (Infinite M200, Tecan, Austria) at 510 nm excitation and 595 nm emission wavelengths. For control of the threshold activity, cells were preincubated for 15 min with 2 μM KCN in complete medium and measurements were performed as described above but in PBS containing 2 μM KCN. The activity values were normalized to mg of protein.

### ATP/ADP ratio

ADP and ATP levels were measured using an ADP/ATP ratio kit (Abcam).

### Sub-cellular fractionation

Sub-cellular fractionation was performed essentially as described [48,49]. Briefly, cells (10^9^) were harvested, washed in PBS, pelleted, resuspended in homogenization buffer (0.25 M sucrose and 10 mM Hepes pH 7.4) and gently disrupted by dounce homogenization. Upon gentle centrifugation to remove cellular debris and nuclei, the supernatant was centrifuged at 10.300 x g for 10 min to pellet crude mitochondria, which were resuspended in isolation medium (250 mM mannitol, 5 mM Hepes pH 7.4, 0.5 mM EGTA).

### Microscopic analysis of mitochondrial structure

Mitochondrial structure was studied after loading 10nM of Tetramethylrhodamine methyl ester (TMRM). Imageswere recorded using a digital imaging system based on a Zeiss Axiovert 200 fluorescence microscope equipped with a back-illuminated CCDcamera (Roper Scientific, USA), excitation and emission filter wheels(Sutter Instrument Company, USA) and piezoelectric motoring of the zstage (Physik Instrumente, GmbH & Co., Germany). The data wereacquired and processed using the MetaFluor analyzing program(Universal Imaging Corporation, USA).

### Small animal PET

PET images were acquired on the positron emission tomograph for small animals YAP-(S)PET system [50]. Mice were fasted overnight before PET acquisition, anesthetized by inhalation of 2% of isofluorane and intravenously injected with 350μCi±50 of [^18^F]fluorodeoxyglucose ([^18^F]FDG) in a 0.15-ml volume. The residual dose in the syringe was measured to verify the effective injected dose. The tumour was centred on the field of view of the tomograph and a static acquisition started after 45 minutes of uptake. A 3D data acquisition mode and an expectation maximization (EM) algorithm with 30 iterations for image reconstruction were used, the resulting voxel size was 0.5×0.5×2mm3.No corrections were made for attenuation and scatter. The images were visualized with dedicated software in the three planes (transaxial, sagittal, and coronal). Quantitative image analysis of tracer uptake was evaluated by drawing region of interest (ROI) of tumour on the transaxial images. ^18^F]FDG uptake was quantified as standardized uptake values (SUV) and as percentage of the injected dose per gram of tissue (%ID/g).

### Statistical analysis

An unpaired *t* test was used to calculate a P value for two groups, while a P value on a response affected by two factors was calculated with a two-way ANOVA [51].

## SUPPLEMENTARY FIGURES

Supplementary Figure S1. Phenotype of the *Stat3^C/C^* MEFs. (**A**) *Stat3^C/C^* MEFs show an accelerated cell cycle. Cells were starved for 24 hours, stimulated with serum and analyzed after the indicated times. The percentage (mean ± s.e.m.) of cells in S-phase was calculated by flow cytometry after staining with propidium iodide and represent three independent samples per genotype. (**B**,**C**) *Stat3^C/C^* MEFs are protected from apoptosis upon serum starvation. Cells plated on coated glass slides were subjected to serum-starvation for 72 hours and then stained with a TUNEL assay (**B**) or with an antibody against activated caspase-3 (**C**). (**B**) Arrows indicate TUNEL-positive cells. Nuclei are shown in blue. Numbers represent the mean percentage of positive cells ± s.e.m. of three independent samples per genotype. (**C**) Numbers represent the percentage (mean ± s.e.m.) of positive cells from three independent samples per genotype. *, p < 0.01. (**D**) Menadione-induced apoptosis. Cells were treated with 7,5 μM Menadione for 24 hours. (**E**) UV-induced apoptosis. Cells were treated with UV light (10 j/m^2^) and stained for Annexin V after 24 hours. (**D,E**) Data represent the percentage of Annexin V positive cells (mean ± s.e.m.) of three independent samples per genotype. *, p < 0.01. Empty bars or filled bars, *Stat3^WT/WT^* or *Stat3^C/C^* MEFs.

Supplementary Figure S2. Decreased expression of mRNAs encoding for mitochondrial proteins in *Stat3^C/C^* MEFs. (**A**) List of mRNAs annotated as ‘mitochondrion’ whose expression was significantly lower in *Stat3^C/C^* MEFs in the microarray experiment of Figure 2. (**B**) Taqman RT-PCR on nuclear-encoded mitochondrial genes. Empty bars or filled bars, *Stat3^WT/WT^* or *Stat3^C/C^* MEFs respectively, either silenced or not for HIF-1α (sh-HIF-1α), represent mean values ± s.e.m. of three independent experiments. *, p ≤ 0,01. ATP-5L, ATP synthase, H+ transporting, mitochondrial F0 complex, subunit G; FH, fumarate hydratase; MRPL39, 39S ribosomal protein L39, mitochondrial.

Supplementary Figure S3. The glycolytic phenotype specifically depends on Stat3 activity. (**A-C**) Inhibition of the PI3-K/AKT pathway. Cells were treated for 48 hours with the PI3 Kinase inhibitor Ly294002. (**A**) Western blot analysis on total protein extracts showing decreased P-AKT upon Ly294002 treatment. The expression of Hif-1α and Pdk1 mRNAs (**B**) and the accumulation of lactate (**C**) are not affected by Ly294002 treatment. (**D,E**) Similar Hif-1α and Pdk1 mRNAs levels and lactate accumulation in *Stat3^+/+^* or *Stat3^-/-^* 3T3 MEFs. Data are shown as mean values ± s.e.m. of three samples per genotype.

Supplementary Figure S4. Stat3-dependent glycolytic metabolism and mitochondrial activity in tumour cell lines. (**A**) The STAT3-dependent MDA-MB468, SKBR3, DU145 and the STAT3-independent T47D human tumour cells were either treated or not with the S3I STAT3 inhibitor for 12 hours followed by the analysis of total and Y705 phosphorylated STAT3 by Western-blot. (**B**) Percentage of Annexin V^+^ MDA-MB468 cells treated with the S3I compound for the indicated times. (**C-E**) Gene expression, lactate production and mitochondrial Ca^2+^ release were measured in the SKBR3 (**C**), DU145 (**D**) and T47D **(E)** cell lines as described in the legends to figures 3 and 4. *, p ≤ 0,01 (n=3).

SUPPLEMENTARY MATERIALS AND METHODS
