# Supplementary figures and images for: A STAT3-mediated metabolic switch is involved in tumour transformation and STAT3 addiction
Source: Aging (Albany NY). 2010 Nov 15;2(11):823–42. doi: 10.18632/aging.100232 (PMC3006025; doi:10.18632/aging.100232)

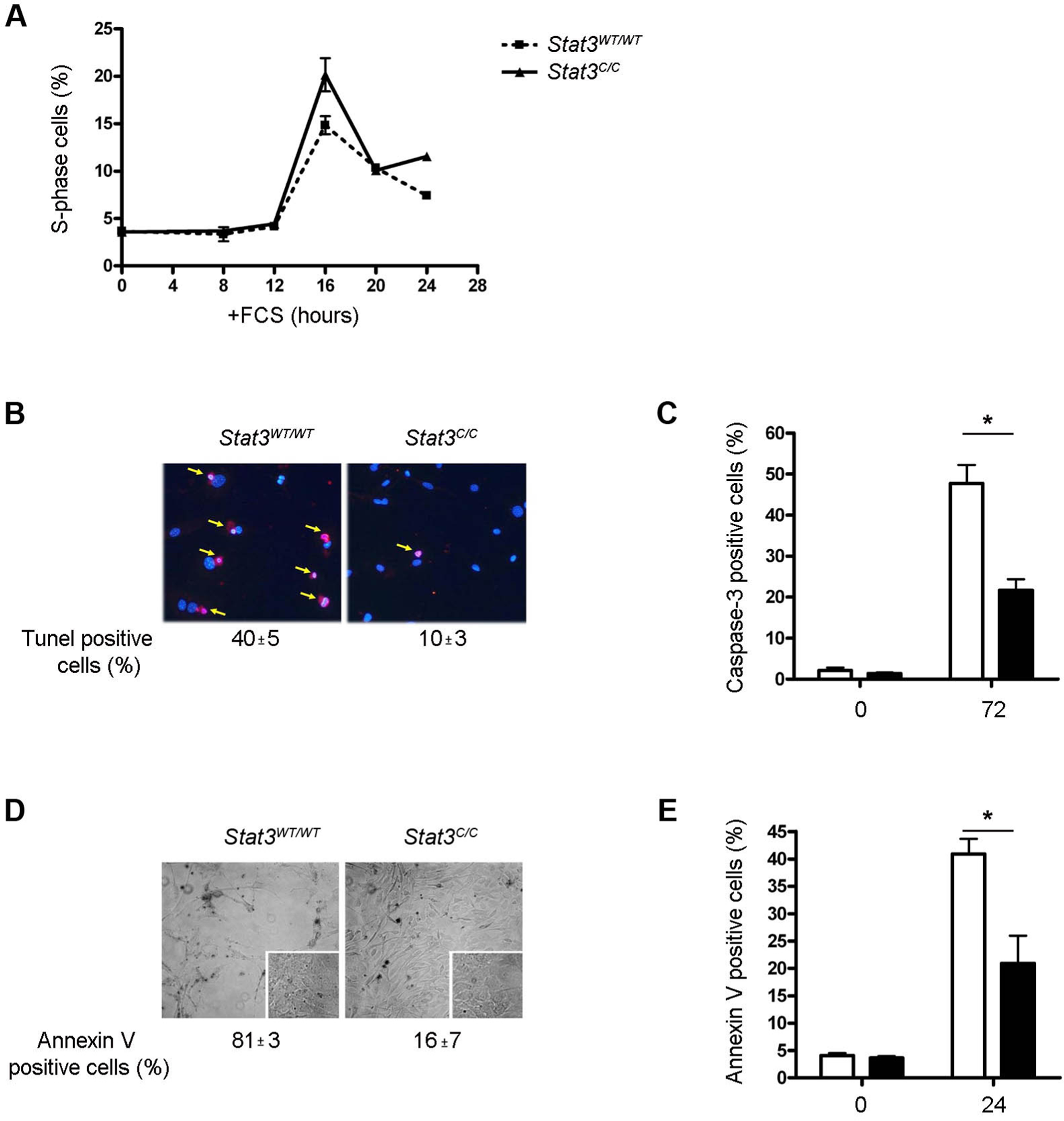

Supplement: Supplementary Figure S1. — Phenotype of the Stat3C/C MEFs. (A) Stat3C/C MEFs show an accelerated cell cycle. Cells were starved for 24 hours, stimulated with serum and analyzed after the indicated times. The percentage (mean ± s.e.m.) of cells in S-phase was calculated by flow cytometry after staining with propidium iodide and represent three independent samples per genotype. (B,C) Stat3C/C MEFs are protected from apoptosis upon serum starvation. Cells plated on coated glass slides were subjected to serum-starvation for 72 hours and then stained with a TUNEL assay (B) or with an antibody against activated caspase-3 (C). (B) Arrows indicate TUNEL-positive cells. Nuclei are shown in blue. Numbers represent the mean percentage of positive cells ± s.e.m. of three independent samples per genotype. (C) Numbers represent the percentage (mean ± s.e.m.) of positive cells from three independent samples per genotype. *, p < 0.01. (D) Menadione-induced apoptosis. Cells were treated with 7,5 μM Menadione for 24 hours. (E) UV-induced apoptosis. Cells were treated with UV light (10 j/m2) and stained for Annexin V after 24 hours. (D,E) Data represent the percentage of Annexin V positive cells (mean ± s.e.m.) of three independent samples per genotype. *, p < 0.01. Empty bars or filled bars, Stat3WT/WT or Stat3C/C MEFs. [file aging-02-823-s001.tif]

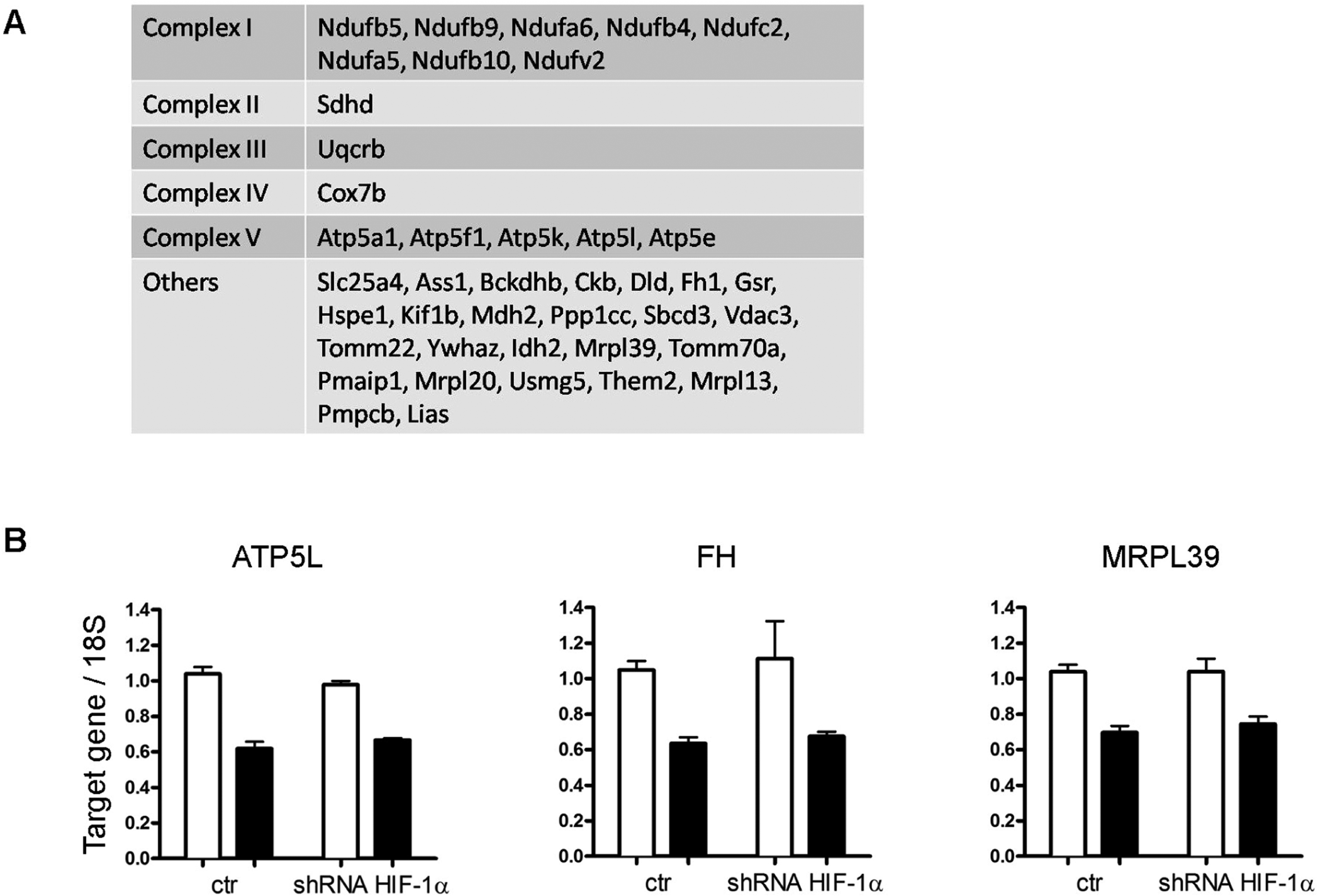

Supplement: Supplementary Figure S2. — Decreased expression of mRNAs encoding for mitochondrial proteins in Stat3C/C MEFs. (A) List of mRNAs annotated as ‘mitochondrion’ whose expression was significantly lower in Stat3C/C MEFs in the microarray experiment of Figure 2. (B) Taqman RT-PCR on nuclear-encoded mitochondrial genes. Empty bars or filled bars, Stat3WT/WT or Stat3C/C MEFs respectively, either silenced or not for HIF-1α (sh-HIF-1α), represent mean values ± s.e.m. of three independent experiments. *, p ≤ 0,01. ATP-5L, ATP synthase, H+ transporting, mitochondrial F0 complex, subunit G; FH, fumarate hydratase; MRPL39, 39S ribosomal protein L39, mitochondrial. [file aging-02-823-s002.tif]

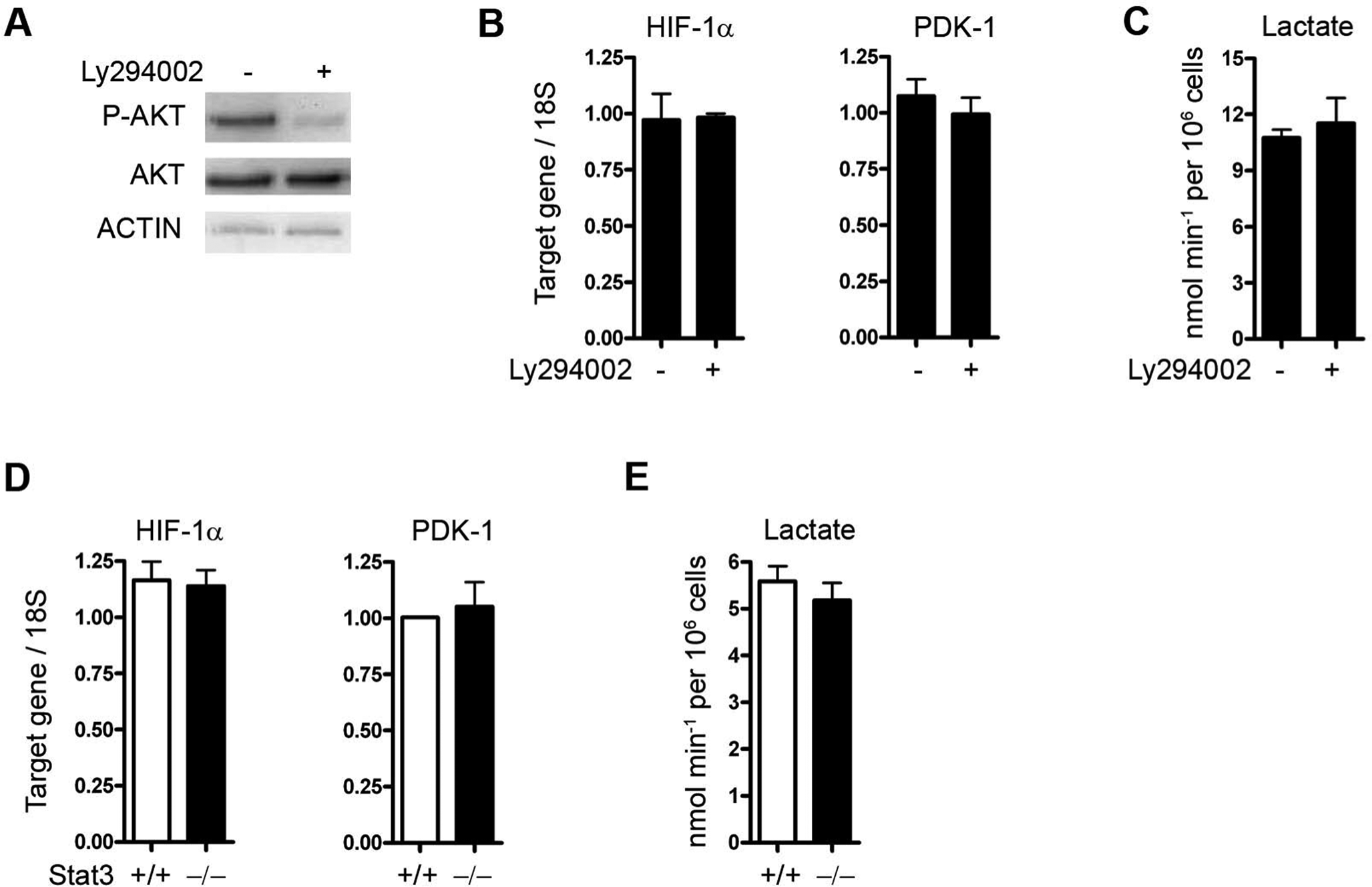

Supplement: Supplementary Figure S3. — The glycolytic phenotype specifically depends on Stat3 activity. (A-C) Inhibition of the PI3-K/AKT pathway. Cells were treated for 48 hours with the PI3 Kinase inhibitor Ly294002. (A) Western blot analysis on total protein extracts showing decreased P-AKT upon Ly294002 treatment. The expression of Hif-1α and Pdk1 mRNAs (B) and the accumulation of lactate (C) are not affected by Ly294002 treatment. (D,E) Similar Hif-1α and Pdk1 mRNAs levels and lactate accumulation in Stat3+/+ or Stat3-/- 3T3 MEFs. Data are shown as mean values ± s.e.m. of three samples per genotype. [file aging-02-823-s003.tif]

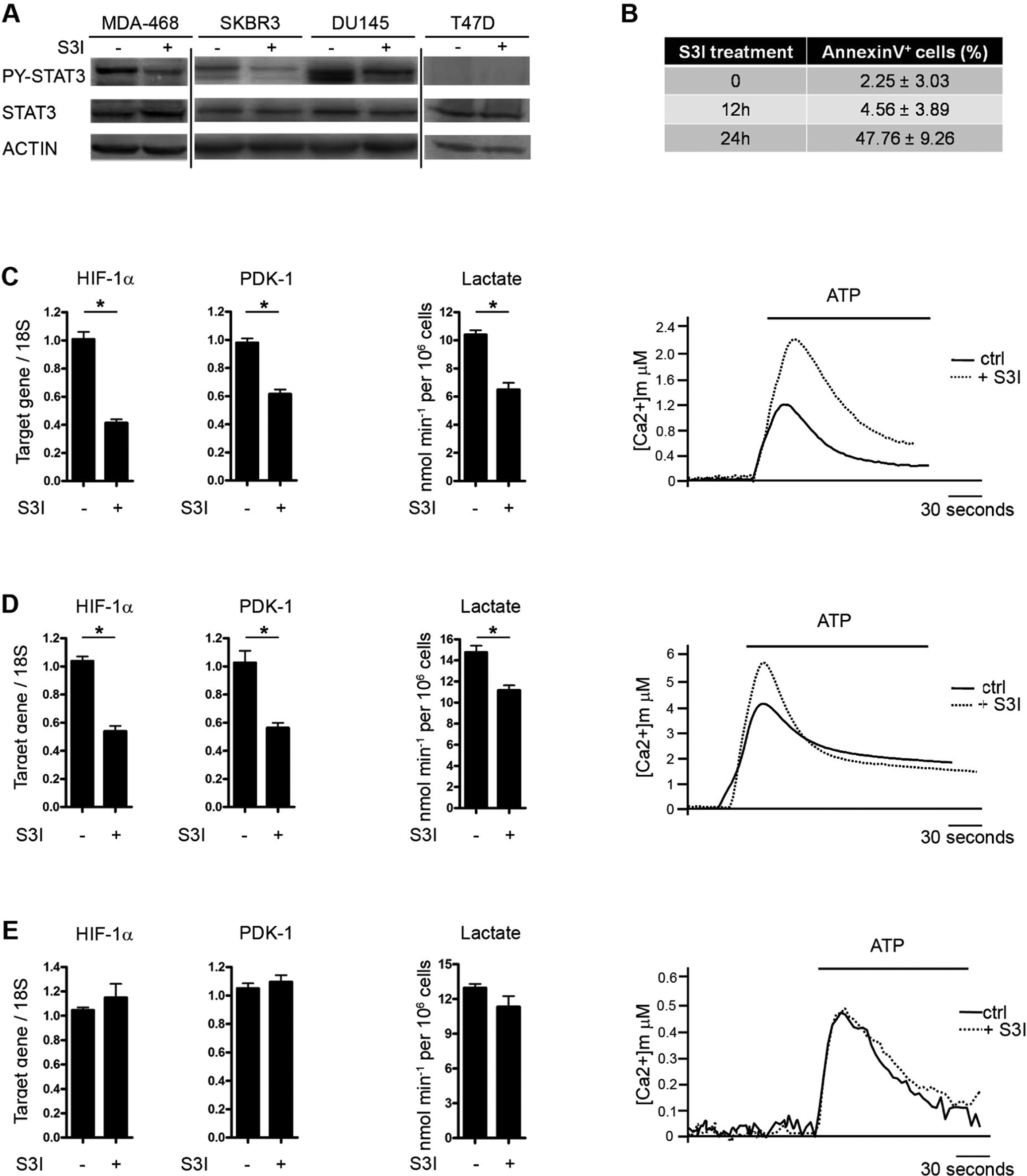

Supplement: Supplementary Figure S4. — Stat3-dependent glycolytic metabolism and mitochondrial activity in tumour cell lines. (A) The STAT3-dependent MDA-MB468, SKBR3, DU145 and the STAT3-independent T47D human tumour cells were either treated or not with the S3I STAT3 inhibitor for 12 hours followed by the analysis of total and Y705 phosphorylated STAT3 by Western-blot. (B) Percentage of Annexin V+ MDA-MB468 cells treated with the S3I compound for the indicated times. (C-E) Gene expression, lactate production and mitochondrial Ca2+ release were measured in the SKBR3 (C), DU145 (D) and T47D (E) cell lines as described in the legends to figures 3 and 4. *, p ≤ 0,01 (n=3). [file aging-02-823-s004.tif]
