# Supplementary material for: A STAT3-mediated metabolic switch is involved in tumour transformation and STAT3 addiction
Source: Aging (Albany NY). 2010 Nov 15;2(11):823–42. doi: 10.18632/aging.100232 (PMC3006025; doi:10.18632/aging.100232)
Supplement: SUPPLEMENTARY MATERIALS AND METHODS [file aging-02-823-s005.doc]

**SUPPLEMENTARY MATERIALS AND METHODS**

Primer sequences and probes used for Taqman PCR:

mmu HIF-1α upper, 5’-gcactagacaaagttcacctgaga-3’

mmu HIF-1α lower, 5’-cgctatccacatcaaagcaa-3’, Probe: #95

mmu PDK-1 upper, 5’-gttgaaacgtcccgtgct-3’

mmu PDK-1 lower, 5’-gcgtgatatgggcaatcc-3’, Probe: #20

mmu GLUT-1 upper, 5’-atggatcccagcagcaag-3’

mmu GLUT-1 lower, 5’-ccagtgttatagccgaactgc-3’, Probe: #52

mmu PFK-L upper, 5’-ccatggacgaggagaggtt-3’

mmu PFK-L lower, 5’-tccagttgttctcaaagctcct-3’, Probe: #42

mmu ENO-1 upper, 5’-gaggcgcttagtgctgct-3’

mmu ENO-1 lower, 5’-agaatagacatggcgaatttctg-3’, Probe: #91

mmu OSM-R upper, 5’- ccaaaaagagttcagcacacc -3’

mmu OSM-R lower, 5’- ccgaccacacttgtctccat -3’, Probe: #32

mmu PDGFRa upper, 5’-gtcgttgacctgcagtgga-3’

mmu PDGFRa lower, 5’-ccagcatggtgatacctttgt-3’, Probe: #80

mmu SOCS-3 upper, 5’-atttcgcttcgggactagc-3’

mmu SOCS-3 lower, 5’-aacttgctgtgggtgaccat-3’, Probe: #83

mmu ATP-5L upper, 5’-aaggaagctgtgctgaatgg-3’

mmu ATP-5L lower, 5’-atgccacgtttgcctatga-3’, Probe: #42

mmu FH-1 upper, 5’-gcaccccaatgatcatgtta-3’

mmu FH-1 lower, 5’-cattgctgtgggaaaggtg -3’, Probe: #106

mmu NDUFB4 upper, 5’-agggtggtgaagtggaagag-3’

mmu NDUFB4 lower, 5’-tggccacttccactggtta-3’, Probe: #31

mmu NDUFA6 upper, 5’-cccactcccaagaactcact-3’

mmu NDUFA6 lower, 5’-tggataagtctttcctttctgtcc-3’, Probe: #17

hsa HIF-1α upper, 5’-ggttcactttttcaagcagtagg-3’

hsa HIF-1α lower, 5’-tggtaatccactttcatccattg-3’, Probe: #3

hsa PDK-1 upper, 5’-gctgggtaatgaggatttgact-3’

hsa PDK-1 lower, 5’-aagtctgtcaattttcctcaaagg-3’, Probe: #10

hsa ENO-1 upper, 5’-gctccgggacaatgataaga-3’

hsa ENO-1 lower, 5’-tgatgtgctcaacagccttt-3’, Probe: #60

Antibodies. Rabbit polyclonal against p-Stat3, p-Akt and Caspase-3 (Cell Signaling Technology, Danvers MA, USA), Stat3 and Actin (Santa Cruz Biotechnology, Santa Cruz CA, USA), Vdac-1 (Abcam, Cambridge, UK). Mouse monoclonal against Hif-1α (Novus Biologicals, Littleton CO, USA), Sod2 (Abcam) and β-Tubulin (Santa Cruz Biotechnology). Mouse monoclonal against Akt was generated in house by immunization with a MBP-Akt fusion protein and purified against the recombinant protein. The MitoProfile total OXPHOS rodent WB Antibody Cocktail (Mitosciences, Eugene OR, USA) was used for mitochondrial complexes.

In vivo model. 8 Female mice 5 to 7 weeks of age were s.c. implanted in CD-1 mice (Charles River Laboratories, Inc.) with MDA-MB468. Cells were harvested using trypsin and washed with PBS. 10^7^ Cells were resuspended in 200 μL PBS and injected s.c. into the left flank of mice. 14 days after the inoculation of tumour cells, mice were randomly divided in two groups and the first PET acquisition was performed. Subsequently the first group (n=5) received in the tail vein a 5 mg/kg of S3I three time per week for 2 consecutive weeks; the second group (n=3) was not treated. All mice were monitored two times weekly for body weight and tumour measurement.
